# The relationship between dietary micronutrients and endometriosis: A case-control study

**DOI:** 10.18502/ijrm.v21i4.13272

**Published:** 2023-05-08

**Authors:** Ghazal Roshanzadeh, Shahideh Jahanian Sadatmahalleh, Ashraf Moini, Azadeh Mottaghi, Farahnaz Rostami

**Affiliations:** ^1^Department of Midwifery and Reproductive Health, Faculty of Medical Sciences, Tarbiat Modares University, Tehran, Iran.; ^2^Department of Obstetrics and Gynecology, Arash Women's Hospital, Tehran University of Medical Sciences, Tehran, Iran.; ^3^Breast Disease Research Center (BDRC), Tehran University of Medical Sciences, Tehran, Iran.; ^4^Department of Endocrinology and Female Infertility, Reproductive Biomedicine Research Center, Royan Institute for Reproductive Biomedicine, ACECR, Tehran, Iran.; ^5^Research Center for Prevention of Cardiovascular Disease, Institute of Endocrinology and Metabolism, Iran University of Medical Sciences, Tehran, Iran.

**Keywords:** Diet, Endometriosis, Food questionary, Micronutrients.

## Abstract

**Background:**

Fewer studies were on micronutrient intake in women with endometriosis, and the etiology of endometriosis remains unclear between dietary micronutrients and the risk of endometriosis.

**Objective:**

This study aimed to investigate the relationship between dietary micronutrients and the risk of endometriosis.

**Materials and Methods:**

This case-control study was conducted on 156 women (18-45 yr) with and without endometriosis in the gynecology clinic of Arash hospital between May 2017 and May 2018 in Tehran, Iran. According to the laparoscopic findings, the participants were divided into 2 groups (n = 78/each), women with pelvic endometriosis as the case group and women without endometriosis pelvic as the control group. Dietary data were collected using a validated 168-item semiquantitative food frequency questionnaire with the standard serving. A logistic regression model was used to determine the association between micronutrients and the risk of endometriosis.

**Results:**

Data analysis showed a significant relationship between micronutrients such as: potassium (OR: 0.74; CI: 0.56-0.99; p = 0.01), calcium (OR: 0.70; CI: 0.52-0.94; p = 0.003), and also among the vitamin C (OR: 0.70; CI: 0.52-0.94; p = 0.02), B2 (OR: 0.73; CI: 0.55-0.98; p = 0.01), and B12 (OR: 0.71; CI: 0.53-0.95; p = 0.02) with endometriosis, so those who used fewer micronutrients were at higher risk of endometriosis.

**Conclusion:**

The findings showed that the dietary intakes of calcium, potassium, vitamins B12, B2, B6, and C are inversely related to the risk of endometriosis.

## 1. Introduction 

Endometriosis is an inflammatory disease of the pelvis characterized by the implantation of endometrial tissue outside the uterine cavity and endometrial tissue outside the cavum uteri. Its prevalence in the world is estimated at 10% (1). The approximate prevalence of endometriosis in women is 6% during the reproductive age (2). Endometriosis is accepted as an estrogen-dependent disease (3). Endometriosis has varying signs and symptoms in terms of severity and include dysmenorrhea, dyspareunia, infertility, dysuria, and dyschezia (4, 5). Despite the prevalence and associated morbidity, the etiology of endometriosis is still poorly understood, and few modifiable risk factors have been identified so far. Diet is one of the main factors influencing endometriosis risk. Diet can act through inflammation, estrogenic effects, immune function, and smooth muscle contractility (4). Some studies have shown associations between the intake of certain nutrients and the pathophysiological mechanisms of endometriosis. Consuming supplements of different antioxidants reduces pelvic pain (6, 7).

The use of vitamins and minerals, constituting the orthomolecular approach (The term “orthomolecular" to characterize the treatment of disease with nutrients that are endogenous to the human body), is successful when taken up by endometriotic subjects, as they can reduce pain endometriosis via anti-inflammatory effects, promote immunity, and prevent endometriosis (8).

Also, a study on the risk of developing endometriosis has shown that consuming enriched nutrients with folate, methionine, vitamins B6, A, C, and E act on the genome by influencing decreased DNA methylation endometriosis. A deficiency of these nutrients in the diet affected alterations in lipid metabolism, like oxidative stress and epigenetic abnormalities (9).

Vitamin B, especially pyridoxine (B6), increases the inactive metabolism of estrogen and supports the conversion of linoleic acid to the gamma-linoleic acid pathway, which is an essential component in the production of anti-inflammatory prostaglandins. These anti-inflammatory prostaglandins can inhibit the growth of endometrial tissue (10).

Moreover, diet can increase oxidative stress, estrogen levels, and prostaglandin metabolism, leading to endometriosis. It can also be as dietary antioxidants such as vitamins A and C contributing to transporting free radicals and active oxygen species, leading to decreased growth and adhesion of endometrial cells in the peritoneal cavity of women with endometriosis (9).

Despite, few studies on micronutrients and the risk of developing endometriosis. Still, some specific diets that affect endometriosis etiopathology, including a lower dietary intake of calcium, which was associated with the risk of endometrioma (10, 11). This study showed the consumption of additional antioxidants, as well as a combination of vitamins and minerals having a positive effect on the symptoms associated with endometriosis (12). This study showed that the consumption of additional antioxidants and a combination of vitamins and minerals have a positive effect on the symptoms associated with endometriosis.

Fewer studies are available on the micronutrient intake of women with endometriosis. Therefore, this study aimed to evaluate the precise diet and micronutrient intake (zinc, copper, folate, phosphorus, magnesium, sodium, potassium, fluoride, calcium, vitamin C, iron, vitamin B, vitamin D, and vitamin E), to analyze the validated food frequency questionnaire (FFQ) and the association between dietary micronutrients and the risk of endometriosis.

## 2. Materials and Methods

### Study population

This case-control study was conducted on 156 women (18-45 yr) with and without endometriosis in the Gynecology Clinic of Arash hospital, Tehran, Iran between May 2017 and May 2018. The case group was allocated to infertile women who underwent diagnostic laparoscopy during the study period.

After surgery, the stage of the disease was defined according to the classification system of the revised American Society for Reproductive Medicine as stages of minimal, mild, moderate, and severe (13).

Women who had abnormalities other than endometriosis at laparoscopy were excluded from the study. The control group was also among women aged between 18-45 who met the inclusion criteria, had secondary dysmenorrhea, and no visible lesions of endometriosis. In terms of age and level of education and employment status, economic conditions, parity, marital status, height, and weight were matched with the case group.

### Sample size

Based on previous study results (14) with 99% confidence and 90% test power, the required number of samples for each group was equal to the following formula: 


$$n=\frac{\left(s_{1}^{2}+s_{2}^{2}\right){\left(z_{1-\frac{\alpha }{2}}+z_{1-\beta }\right)}^{2}}{{\left({\bar{x}}_{1}-{\bar{x}}_{2}\right)}^{2}}$$

The sampling method was as follows: 69 people were determined for both case and control groups, with calculated 10% drop, 78 people in each group and 156 people were determined. A convenient sampling technique was used.

The subjects were selected from the patients referred to the laparoscopic ward of Arash Women's Comprehensive hospital, if they were eligible to enter our study, with their knowledge and consent. The FFQ was filled out for study.

Overall, 156 women were included in the study. The laparoscopy findings were divided into 2 groups: 78 women with endometriosis (case group) and 78 women with a normal pelvis without endometriosis (control group). The women in the 2 groups were comparable in demographic and personal characteristics. Inclusion criteria were as follows: 1) women age between of 18-45 yr, 2) not pregnant, 3) Iranian race, 4) absence of a history of chronic diseases, 5) not using medication affecting the adsorption of nutrients, food intake, appetite, and basal metabolism of the body, 6) lack of mental retardation, and 7) no smoking. Exclusion criteria: 1) incomplete filling of FFQ and 2) people with more than 6500 calories.

This study matched groups in terms of age, body mass index (BMI), parity, severity, marital status, economic status, and education level.

### Anthropometrics and lifestyle measurements

Trained interviewers collected the participant's characteristics using a questionnaire. Information on age (years), educational level, marital status, use of hormones, anti-inflammatory drugs, associated comorbidities, income, age at menarche, parity, and employment status were also assessed.

The subjects were minimally clothed without shoes, using digital scales to the nearest 100-gram their weight was measured. Height was measured to the nearest 0.5 cm in a standing position without shoes using a tape meter. BMI was calculated as weight (kg) divided by the square of the height (m^2^).

### Assessment of dietary intake

Using a validated semiquantitative FFQ with 168 food items dietary data were collected. The Persian version of FFQ has previously been evaluated for reliability and validity (15). An experienced researcher completed all questionnaires. The FFQ contains a list of common food items with standard size (unit) or the most commonly familiar amount to people in this community. The participants were asked for each food item in the questionnaire, to report their consumption. Over the past year per day, week, or month. The reported frequency was converted to daily intake based on the unit size for each food item. The values of home modules were used to convert the serving size of consumables to grams. In terms of the amount of energy and nutrients, every food and drink were analyzed using Nutritionist 4 software (Professor P. Mirmiran, Tehran, Iran).

### Ethical considerations

The study has been approved by the Ethics Committee of Tarbiat Modares University of Medical Sciences, Tehran, Iran (Code: IR.TMU.REC.1395.359). Also, a signed written consent was obtained from all participants.

### Statistical analysis

Statistical analysis of the obtained data was performed by using Statistical Version 23, SPSS Inc., Chicago, Illinois, USA (SPSS). Using Kolmogorov-Smirnov's test. The normality of socio-demographic characteristics was checked. Using the *t* test and Mann-Whitney Comparisons between the 2 groups were done, and Chi-square tests, and P-value < 0.05 was considered statistically significant. For the different variables, odds ratio (OR; adjusted for age, total energy intake, BMI, and income) and 95% confidence intervals (CIs) were calculated using logistic regression models to assess the strength of the associations between the intake micronutrient and the risk of endometriosis. Each dietary variable and micronutrient were further divided into 4 groups using a quartile of intake based on the distribution of control subjects. To calculate the linear trend in the odds of the dietary variable quartile, the median factor score of each quartile was entered into the logistic regression analysis. Quartile 1 served as the reference category for all regression analyses.

## 3. Results

The anthropometric and demographic characteristics of the 156 women in case and control groups were collected. The severity of the disease was staged according to the revised American Society for Reproductive Medicine classification of endometriosis. Endometriosis was staged as minimal (stage I) in 3 (3.8%), mild (stage II) in 7 (8.9%), moderate (stage III) in 25 (32.0%), and severe (stage IV) in 43 (55.1%).

Total energy using the residual method, to control for total energy for all nutrients intake was adjusted. The estimated energy intake was 2443.31 ± 608.21 kcal/day for the women in the endometriosis group and 2566.42 ± 607.59 kcal/day for those in the control group.

No significant difference was observed between the 2 groups in age, age at menarche, parity, marital status, income, BMI, education level, employment status, and daily calorie intake. Women over 35 had the highest percentage of age in the groups (Table I).

Table II showed the micronutrient intake in the case and control groups and the recommended levels for each micronutrient. A significant difference was observed between the vitamins total folate (p = 0.04), and vitamin B6 (p = 0.04) in the case and control groups. Thus, the consumption of total folate and vitamin B6 nutrients in the case group was less, than that in the control group. However, no significant difference was observed between niacin, thiamine, vitamin B7, and vitamin B5 in the 2 groups (p < 0.05).

Also, the results showed a statistically significant difference between the case and control groups in vitamin B12 (p = 0.02), riboflavin (p = 0.01), and vitamin C (p = 0.02).

Minerals including zinc (p = 0.04), iron (p = 0.04), and potassium (p = 0.01) showed a statistically significant difference. The diet indicates lower consumption of these elements in the case group than in the control group (Table II).

Table III summarizes the odds ratio for endometriosis by mineral intake according to the quartile of intake. We observed inverse associations between endometriosis risk and the consumption of potassium (OR: 0.74; 95% CI: 0.56-0.99; P-trend = 0.01) and calcium (OR: 0.70; 95% CI: 0.52-0.94; P-trend = 0.003).

Table IV demonstrates the quartiles of daily food intake and the OR of endometriosis with the corresponding 95% CIs according. High consumption of riboflavin (B2) (OR: 0.69; 95% CI: 0.51-0.92; P-trend = 0.01), vitamin B6 (OR: 0.73; 95% CI: 0.55-0.98; P-trend = 0.04), vitamin B12 (OR: 0.71; 95% CI: 0.53-0.95; P-trend = 0.04), vitamin C (OR: 0.70; 95% CI: 0.52-0.94; P-trend = 0.02), and Beta-carotene (OR: 0.70; 95% CI: 0.53-0.94; P-trend = 0.02) were associated with a lower risk of endometriosis. Increased consumption of vitamin E was associated with a reduced risk of endometriosis (second quartile OR: 0.32; %95 CI: 0.10-0.82, third quartile OR: 0.26; %95 CI: 0.10-0.67; P-trend = 0.09).

**Table 1:** Anthropometric and demographic characteristics of their case and control groups


**Characteristics**		**Case group (n = 78)**	**Control group (n = 78)**	**P-value**
**Age (yr) **		31.24 ± 6.63	29.94 ± 6.86	0.23*
**Menarche age (yr)**		13.49 ± 2.38	13.35 ± 1.64	
**Median (IQR)**		13 (12-15)	13 (12-15)	0.70**
**Parity**				
	**Parous**	32 (41)	42 (54.6)	
	**Nulliparous**	46 (59)	35 (45.5)	0.06***
**Marital status**				
	**Single**	22 (28.2)	19 (24.4)	
	**Married**	56 (71.8|)	59 (75.6)	0.58***
**Income (million Tomans)**				
	**< 1 **	24 (30.8)	21 (26.1)	
	**Between 1 and 2 **	33 (42.2)	34 (34. 4)	
	**Between 2 and 2.5 **	8 (10.3)	13 (16.7)	
	**> 2.5 **	13 (16.7)	10 (12.8)	0.61***
**BMI (kg/m^2^)**				
	**Thin**	10 (12.82)	10 (12.82)		
	**Normal**	36 (46.15)	35 (44.87)		
	**Overweight **	29 (37.17)	27 (34.61)	
	**Obese **	3 (3.84)	6 (7.69)	0.78***
**Education**				
	**Lower than university **	42 (53.8)	38 (49.4)		
	**University**	36 (46.2)	39 (50.6)		0.58***
**Employment status**					
	**Housewife **	61 (78.2)	68 (88.3)	
	**Employed**	17 (21.8)	9 (11.7)	0.09***
**Daily calorie intake (kcal/d) **		2443.31 ± 608.21	2566.42 ± 607.59		
**Median (IQR)**		2443.70 (1994.12-2895.08)	2547.82 (2077.71-2949.88)	0.20**
*Values are given as Mean ± SD. Student's *t* test, **Values are given as Mean ± SD. Mann-Whitney test, ***Values are given as number (%). Chi-squared test, BMI: Body mass index, IQR: Interquartile range				

**Table 2:** Comparison of micronutrient intake between groups


	**Case group (n = 78)**		**Control group (n = 78)**		
**Nutrient item (vitamins)**	**Median (IQR)**	**Mean ± SD**	**Median (IQR)**	**Mean ± SD**	**P-value**
	**Vitamin A (µg)**	567.42 (390.91-729.35)	663.17 (405.09-804.01)	0.23*	
**Vitamin E (α-tocopherol)**	13.91 (10.24-19.44)	15.24 (12.46-17.52)	0.09*
**Vitamin K (µg)**	339.50 (169.03-423.91)	369.68 (217.70-672.44)	0.70*
**Vitamin D (µg)**	1.25 (0.62-2.34)	1.52 (0.73-2.85)	0.12*
**Thiamin (B1) (mg)**	2.04 (1.75-2.51)	2.12 (1.72-2.67)	0.20
**Riboflavin (B2) (mg)**	2.05 (1.70-2.40)	2.30 (1.81-2.93)	0.01*
**Niacin (B3) (m)**	- 25.12 ± 6.81	- 26.31 ± 6.87	0.28**
**Pantothenic acid (B5) (mg)**	- 5.28 ± 1.49	- 5.74 ± 1.57	0.05**
**Vitamin B6 (mg)**	- 2 ± 0.60	- 2.19 ± 0.58	0.04**
**Biotin (B7) (µg)**	29.30 (22.70-39.46)	31.81 (24.71-40.57)	0.29*
**Folate (B9) (µg)**	- 594.19 ± 148.52	- 646.35 ± 167.96	0.04**
**Cobalamin (B12) (µg) **	3.32 (2.65-4.44)	4.14 (2.85-5.14)	0.02*
**Vitamin C (mg)**	143.43 (106.62-195.41)	171.36 (127.66-228.21)	0.02*
**Calcium (mg)**	- 1249.3 ± 482.88	- 1502.10 ± 546.56	0.003**
**Chromium (µg)**	0.10 (0.05-0.18)	0.08 (0.03-0.15)	0.18*
**Copper (µg)**	1.79 (1.45-2.27)	1.93 (1.63-2.39)	0.16*
**Fluoride (mg)**	1816.24 (1013.49-2667.74)	1863.27 (1021.69-2688.95)	0.53*
**Iron (mg)**	35.21 (23.56-45.41)	39.40 (27.14-60.50)	0.04*
**Magnesium (mg)**	419.17 (330.71-551.36)	478.41 (356.44-575.01)	0.10*
**Manganese (mg)**	8.46 (6.12-11.53)	9 ± 3.58	8.83 (6.48-11.65)	9.48 ± 3.72	0.44*
**Phosphorus (mg)**	- 1502.29 ± 447.40	- 1671.1 ± 482.25	0.02**
**Potassium (mg)**	4011.14 (3250.71-4822.76)	4778.84 (3508.42-5899.30)	0.01*
**Selenium (µg)**	121.07 (93.47-158.93)	123.25 (94.98-154.43)	0.72*
**Sodium (mg)**	4621.91 (3435.40-6028.16)	4199.41 (3195.87-5729.72)	0.15*
**Zinc (mg)**	11.68 (9.67-14.53)	13.42 (10.53-16.60)	0.04*
**Beta-carotene (µg)**	3109.90 (2134.18-5255.47)	4197.75 (2921.31-5866.81)	0.02*
*Mann-Whitney test. **t test. IQR: Interquartile range					

**Table 3:** Adjusted odds ratios of endometriosis and corresponding 95% CI according to minerals intake in case and control groups


	**Quartile**					
**Nutrient item**	**1**	**2**	**3**	**4**	**Total**	**P-trend**
	**Potassium (mg) **	1 16/23	0.62 (0.25-1.53) 20/19	0.93 (0.37-2.33) 16/23	0.32 (0.12-0.82) 26/13		0.74 (0.56-0.99)	0.01
**Sodium (mg) **	1 18/11	1.21 (0.49-2.98) 24/15	0.50 (0.20-1.25) 16/24	0.72 (0.29-1.79) 20/18	0.83 (0.62-1.1)	0.15
**Calcium (mg) **	1 19/11	1.92 (0.76-4.87) 12/27	0.69 (0.28-1.7) 21/17	0.42 (0.17-1.07) 26/13	0.70 (0.52-0.94)	0.003
**Chromium (µg) **	1 23/16	1.23 (0.50-3.02) 21/18	2.20 (0.88-5.48) 15/23	1.67 (0.68-4.11) 18/21	1.23 (0.93-1.64)	0.18
**Copper (µg) **	1 17/22	0.62 (0.25-1.53) 19/20	0.62 (0.25-1.53) 21/18	0.76 (0.31-1.88) 21/18	0.85 (0.64-1.13)	0.16
**Fluoride (mg) **	1 19/20	(0.42-2.57) 18/21	0.81 (0.33-1.98) 21/19	0.81 (0.32-1.99) 20/18	0.91 (0.68-1.21)	0.53
**Iron (mg) **	1 19/11	1.37 (0.55-3.37) 15/24	0.73 (0.30-1.79) 21/18	0.55 (0.22-1.38) 23/15	0.79 (0.59-1.05)	0.40
**Magnesium (mg) **	1 16/21	0.94 (0.38-2.32) 17/22	0.53 (0.21-1.32) 23/17	0.58 (0.23-1.45) 21/17	0.8 (0.60-1.07)	0.10
**Manganese (mg) **	1 19/21	0.77 (0.31-1.88) 20/18	0.81 (0.33-1.98) 20/19	0.90 (0.37-2.19) 19/20	0.97 (0.73-1.29)	0.44
**Phosphorus (mg) **	1 16/21	0.94 (0.38-2.32) 17/22	0.65 (0.26-1.6) 21/19	0.47 (0.19-1.18) 23/15	0.77 (0.57-1.02)	0.20
**Selenium (µg)**	1 19/20	0.90 (0.37-2.18) 20/20	0.69 (0.28-1.7) 22/17	1.11 (0.45-2.74) 17/21	1.00 (0.75-1.33)	0.72
**Zinc (mg) **	1 17/23	0.81 (0.33-1.98) 18/21	0.66 (0.27-1.61) 20/19	0.45 (0.18-1.12) 23/15	0.77 (0.58-1.03)	0.40
Data presented as OR (95% CI) Case/Control						

**Table 4:** Adjusted odds ratios of endometriosis and corresponding 95% CI according to vitamins intake in case and control groups


	**Quartiles**					
**Nutrient item**	**1**	**2**	**3**	**4**	**Total**	**P-trend**
	**Vitamin E (mg) **	1 12/27	0.32 (0.10-0.82) 22/16	0.26 (0.10-0.67) 25/15	0.49 (0.19-1.25) 18/20		0.91 (0.68-1.21)	0.09
**Vitamin K (µg)**	1 17/23	0.73 (0.29-1.79) 19/20	0.69 (0.28-1.71) 19/19	0.48 (0.19-1.19) 22/17	0.78 (0.58-1.04)	0.07
**Vitamin D (µg) **	1 19/21	1.46 (0.59-3.65) 14/24	0.70 (0.28-1.70) 22/18	0.55 (0.22-1.38) 23/15	0.78 (0.58-1.04)	0.12
**Thiamin (B1) (mg) **	1 21/18	1.43 (0.58-3.52) 17/22	1.29 (0.52-3.17) 18/21	0.85 (0.35-2.10) 22/17	0.94 (0.71-1.25)	0.21
**Riboflavin (B2) (mg) **	1 17/22	1.09 (0.44-2.69) 16/24	0.8 (0.32-1.99) 18/20	0.32 (0.12-0.82) 27/12	0.69 (0.51-0.92)	0.01
**Niacin (B3) (mg) **	1 19/20	1.21 (0.49-2.97) 17/23	0.62 (0.25-1.54) 23/16	0.9 (0.36-2.21) 19/19	0.9 (0.68-1.20)	0.28
**Vitamin B6 (mg) **	1 15/25	0.48 (0.19-1.19) 21/18	0.69 (0.27-1.72) 17/21	0.31 (0.12-0.79) 25/14	0.73 (0.55-0.98)	0.04
**Biotin (B7) (µg) **	1 17/23	0.69 (0.28-1.71) 19/19	0.62 (0.25-1.53) 21/19	0.56 (0.22-1.39) 21/17	0.83 (0.62-1.10)	0.29
**Vitamin B12 (µg) **	1 17/23	0.95 (0.38-2.36) 16/22	0.73 (0.29-1.79) 19/20	0.34 (0.13-0.87) 26/13	0.71 (0.53-0.95)	0.02
**Folate (B9) (µg) **	1 19/20	1.5 (0.60-3.70) 15/25	0.81 (0.32-1.99) 20/18	0.56 (0.22-1.39) 24/15	0.79 (0.59-1.05)	0.11
**Vitamin C (mg) **	1 15/25	0.58 (0.23-1.46) 21/18	0.56 (0.22-1.39) 15/23	0.31 (0.12-0.79) 18/21	0.70 (0.52-0.94)	0.02
**Beta -carotene (µg) **	1 14/26	0.47 (0.19-1.18) 20/19	0.42 (0.17-1.07) 21/18	0.32 (0.12-0.82) 23/15	0.70 (0.53-0.94)	0.02
Data presented as OR (95% CI) Case/Control						

## 4. Discussion 

The findings of this study indicate that phosphorus, total folate, calcium, and vitamin B6 were also normal elements consumed less in the case group than in the control group. In contrast, zinc, iron, potassium, and vitamin B12, riboflavin, and vitamin C were significant in both groups (p < 0.05). The results indicate that these micronutrients were consumed more in the control group than in the case group. Other micronutrients, including sodium, vitamin K, biotin, chromium, fluoride, manganese, magnesium, copper, selenium, vitamin E, and vitamin D showed no significant difference between the 2 groups.

Higher intake of potassium (OR = 0.32, 95% CI = 0.12-0.82; P-trend = 0.01) was not associated with an increased risk of endometriosis, but in people with high potassium intake compared to low consumption was associated with a reduced risk of endometriosis and (OR = 0.7, 95% CI = 0.52-0.92; P-trend = 0.003) total calcium intake was associated with an increased risk of endometriosis.

The main finding of this study is that consumption of an appropriate number of micronutrients like vitamin C, vitamins B (B2, B6, and B12), and potassium can reduce the risk of endometriosis. Among vitamins, vitamins C and E have antioxidant properties and are associated with cell proliferation in response to chronic inflammation and reactive oxygen species (ROS) in endometriosis. Those may play an essential role in cell development and proliferation have reduced endometriosis. The antioxidant activity of these vitamins may suppress the clinical consequences of endometriosis (16, 17). Therefore, they can be safely and efficiently used in treating endometriosis (18). In a high-antioxidant diet, these observations were also seen in vitamins A, C, and E (19). However, in some studies, no significant difference was observed by using different antioxidants in endometriosis (20, 21). But another study found that in women with endometriosis who have chronic pelvic pain, vitamin E and vitamin C supplementation reduced chronic pelvic pain in women with endometriosis, and supplementation of nutritional medicines that interfere with inflammatory processes may benefit women with endometriosis (22). Antioxidants, including vitamin C, vitamin E, vitamin A, selenium, and zinc may affect the endometriosis operating system by upsetting the balance between ROS production and antioxidant levels (23, 24). According to other studies, this study also had different results. Vitamin C was associated with a reduced risk of endometriosis; however, no significant difference was observed between vitamin E and endometriosis in the case and control groups. Vitamin C as an antioxidant reduces the volume of endometriosis lesions in rats. This study supports the use of vitamin C to treat endometriosis (25).

Reportedly, B vitamins (B2, B6, and B12) have reduced the risk of endometriosis. Additionally, they are useful in the treatment of endometriosis (26). B-complex vitamins are generally involved in cell metabolism, increasing the body's metabolism, and increasing cell growth and division. Also, vitamin B6 is one of the group of foods called lipotropic that can alter gene expression or even DNA methylation in humans, decreasing the risk of developing endometriosis (27). Performing regular aerobic exercise with vitamin B6 consumption may decrease *GATA2* gene expression levels of endometriosis in model rat (28). Vitamin B6 helps control endometriosis and improves the level of this disease (29). Also, there was a significant relationship between menstrual pain reduction and vitamin B12 intake (30). However, in some studies, no significant difference was observed between the risk of endometriosis and the intake of vitamins in group B (31). The present study also showed a substantial difference between vitamins B6, B12, B2, and endometriosis. So, less was consumed in the case group than in the control group.

In the minerals that are part of micronutrients, calcium is also associated with endometriosis. Vitamin D increases calcium absorption in the body, and hormones secreted by the thyroid gland regulate calcium levels. The property of calcium in muscle contraction can prevent retrograde menstruation, which is one of the possible causes of endometriosis. Some studies have shown that the factors that reduce the contraction and relaxation of smooth muscles are associated with retrograde menstruation and endometriosis (10). Yet, a direct link between vitamin D levels and predisposition to endometriosis or severity of the disease has not been defined. Compared with controls, some studies in endometriosis patients reported no changes in vitamin D levels. In contrast, other studies showed a significant increase or decrease in vitamin D concentration in women with endometriosis (31). The present study, as in some other studies, had different results. Vitamin D was not significantly different between the 2 groups with and without endometriosis and total calcium intake was associated with an increased risk of endometriosis.

So far, no study has been reported to investigate the association of potassium with endometriosis; however, given the role of this ion on the immune system, it can affect endometriosis. Changes in the level of immune cells, including the level of helper T cells, show women with endometriosis. In this study people with high potassium intake compared to low consumption were associated with a reduced risk of endometriosis (32).

### Limitations

Unfortunately, due to a lack of access to the blood sample of the subjects, blood levels were not measured and reported. More than 90% of the subjects had grade 3 or 4 of the disease, and there was no possibility of examining these individuals in different grades.

## 5. Conclusion

The dietary micronutrient intake of women with endometriosis was lower than healthy women in this study, including zinc, calcium, potassium, and phosphorus in the case group.

Regarding vitamins, vitamin B12, B6, B9, riboflavin, and vitamin C were significant, which showed that the consumption of these vitamins was lower in the case group than in the control group. Other micronutrients such as sodium, iron, thiamine, biotin, chromium, fluoride, manganese, selenium, and vitamin E did not show a significant difference between the 2 groups. It is better to conduct more studies on micronutrients.

##  Conflict of Interest

The authors declare that they have no conflict of interest.
